# Editorial: Data mining and statistical methods for knowledge discovery in diseases based on multimodal omics, volume II

**DOI:** 10.3389/fgene.2023.1270862

**Published:** 2023-08-24

**Authors:** Tao Wang, Miguel E. Rentería, Zhen Tian, Jiajie Peng

**Affiliations:** ^1^ School of Computer Science, Northwestern Polytechnical University, Xi’an, China; ^2^ Key Laboratory of Big Data Storage and Management, Ministry of Industry and Information Technology, Northwestern Polytechnical University, Xi’an, China; ^3^ Mental Health and Neuroscience Program, QIMR Berghofer Medical Research Institute, Brisbane, QLD, Australia; ^4^ School of Computer and Artificial Intelligence, Zhengzhou University, Zhengzhou, China

**Keywords:** multimodal, omics, disease biology, data mining, statistical methods

## Introduction

Thanks to the development of multi-omics technologies, scientists can now accurately measure and analyse numerous biological data modalities simultaneously. Whether these measurements stem from a single study or are integrated from various studies, the rapid accumulation of multimodal omics data, from the bulk-omics to the single-cell omics and spatial omics, is underway. These strides provide an unprecedented opportunity to uncover new knowledge about complex human diseases, through the uncovering of biomarkers, pathways, biological modules, and dynamic molecular networks. These knowledge, in turn, holds potential to enhance therapeutic strategies.

## Current challenges and opportunities

Traditional statistical methods, including transcriptome-wide association studies (TWAS), molecular quantitative trait loci (QTL) analysis, co-localization analysis, or summary-based two-sample Mendelian Randomization, have successfully incorporated multi-omics datasets. However, limitations of these methods have become apparent. Restricted by pre-defined modalities, these methods have hampered the full potential of the diverse omics data available. They can also merely adequately integrate different features, both of which hinder progress in knowledge discovery. Thus, we have organized a Research Topic to showcase the progress of data mining and machine learning methods and discoveries in complex diseases (Wang et al.). The inaugural issue offered a rich tapestry of ideas and innovations, demonstrating both the potential and dynamism of this research field and the enthusiasm of the academic community. Considering the rapid evolution and expansion of the field of multi-omics research, there is a critical need for continued dialogue and exploration. Therefore, here we present the “Volume II” Research Topic. In this Research Topic, we have collated insights on statistical methods and applications geared towards the integration of multimodal omics data, machine learning techniques for characterizing features, and databases coupled with online tools for storing and illustrating disease-related information extracted from multimodal omics. We also ventured into the identification of molecular biomarkers for complex diseases. Through peer-review, eight exceptional articles were published in this Research Topic.

Liu et al. used proteomic analysis to identify potential diagnostic biomarkers for relapse-remitting multiple sclerosis (MS) patients using serum and cerebrospinal fluid. They found 73 differentially expressed proteins in the cerebrospinal fluid and 22 in the serum. Importantly, *MMP2*, *C8G*, and *CFH* were identified as having a high expression trend in both the cerebrospinal fluid and serum, indicating that they may serve a function in the pathogenesis of RRMS.

Gao et al. introduced SpatialMap, a computational method that combines single-cell RNA sequencing data with image-based transcriptomics data to infer gene profiles not previously measured in spatial transcriptomic data. Using generalized linear spatial models, this tool accommodates the count-based nature of spatial gene expression data and considers the spatial correlation among various locations using a conditional autoregressive prior. The authors demonstrated its ability to infer gene expression profiles that have not been measured, across diverse species and technologies.

Sun et al. have proposed a computational framework for predicting MS-associated microRNAs. By using network representation learning techniques and deep learning methods, they aim to improve the prediction of MS-related miRNAs, which could be important biomarkers for the disease. Evaluation of the model demonstrated promising performance, significantly outperforming several existing methods.

The paper by Chen et al. offered a method to explore the evolution of lung adenocarcinoma across different stages. The technique employs a random walk algorithm and the Monte Carlo method to create clusters of biological molecules that show differential expression at each stage of the disease. Through these evolutionary analyses, the suggested method pinpointed 12 core modules and 11 fundamental biological functions.

Ping et al. explored the causal impact of tanning response due to Sun exposure on different skin diseases, employing two-sample Mendelian randomization (MR) for this purpose. They found a positive correlation between the tanning response and the development of six specific skin diseases based on cohorts of European descent, providing a potential link for therapeutic targeting and prevention.

Shi et al. developed a method for predicting the risk of sepsis based on transcriptomic data of patients. The approach involves processing of high-throughput sequencing transcriptomic data and gene annotation, application of machine learning models for forecasting, and utilization of the Shapley Additive explanation (SHAP) method for model interpretation. The most effective machine learning model for predicting sepsis risk was identified as a combination of CatBoost and SHAP.

Liu et al. proposed a machine learning approach to anticipate phosphorylation sites in protein sequences of SARS-CoV-2. This method, based on ensemble learning, extracts features from protein sequences, assesses the significance score of each feature, and utilizes the subset of crucial features to predict phosphorylation sites. This method aids in elucidating the mechanisms of SARS-CoV-2 infection.

Cheng et al. conducted a pan-cancer analysis to investigate the role of the STAT gene family in prognostic prediction and therapeutic guidance. They discovered a variation in the expression of STAT1 between normal and BRCA tissues and identified correlations between STAT expression and multiple characteristics, including immune subtypes, tumor purity, tumor stemness, immune infiltration, immunotherapy response, tumor mutation burden, and drug sensitivity. These findings indicate that STATs could potentially be used as biomarkers for predicting prognosis and guiding therapy in a broad spectrum of cancers.

At least two professional domain experts were required to peer-review all the submissions in this Research Topic. As we reach the conclusion of this Research Topic, we would like to express our sincere appreciation to all authors for their invaluable contributions, and to our diligent readers for their engagement and feedback. The diverse range of research presented has furthered our understanding of the complexities and nuances involved in integrating multimodal data for disease exploration and other trait investigations. Moreover, we express our deep gratitude towards our panel of reviewers, whose expertise and critical insights have ensured the quality and scientific rigor of the published work. We trust that the knowledge shared and generated within this issue will stimulate further innovation and foster continued dialogue in this critical field.

